# Oscillations via Spike-Timing Dependent Plasticity in a Feed-Forward Model

**DOI:** 10.1371/journal.pcbi.1004878

**Published:** 2016-04-15

**Authors:** Yotam Luz, Maoz Shamir

**Affiliations:** 1 Department of Physiology and Cell Biology Faculty of Health Sciences, Ben-Gurion University of the Negev, Beer-Sheva, Israel; 2 Zlotowski Center for Neuroscience, Ben-Gurion University of the Negev, Beer-Sheva, Israel; 3 Department of Physics Faculty of Natural Sciences, Ben-Gurion University of the Negev, Beer-Sheva, Israel; Research Center Jülich, GERMANY

## Abstract

Neuronal oscillatory activity has been reported in relation to a wide range of cognitive processes including the encoding of external stimuli, attention, and learning. Although the specific role of these oscillations has yet to be determined, it is clear that neuronal oscillations are abundant in the central nervous system. This raises the question of the origin of these oscillations: are the mechanisms for generating these oscillations genetically hard-wired or can they be acquired via a learning process? Here, we study the conditions under which oscillatory activity emerges through a process of spike timing dependent plasticity (STDP) in a feed-forward architecture. First, we analyze the effect of oscillations on STDP-driven synaptic dynamics of a single synapse, and study how the parameters that characterize the STDP rule and the oscillations affect the resultant synaptic weight. Next, we analyze STDP-driven synaptic dynamics of a pre-synaptic population of neurons onto a single post-synaptic cell. The pre-synaptic neural population is assumed to be oscillating at the same frequency, albeit with different phases, such that the net activity of the pre-synaptic population is constant in time. Thus, in the homogeneous case in which all synapses are equal, the post-synaptic neuron receives constant input and hence does not oscillate. To investigate the transition to oscillatory activity, we develop a mean-field Fokker-Planck approximation of the synaptic dynamics. We analyze the conditions causing the homogeneous solution to lose its stability. The findings show that oscillatory activity appears through a mechanism of spontaneous symmetry breaking. However, in the general case the homogeneous solution is unstable, and the synaptic dynamics does not converge to a different fixed point, but rather to a limit cycle. We show how the temporal structure of the STDP rule determines the stability of the homogeneous solution and the drift velocity of the limit cycle.

## Author Summary

Oscillatory activity in the brain has been described in relation to many cognitive states and tasks, including the encoding of external stimuli, attention, learning and consolidation of memory. However, without tuning of synaptic weights with the preferred phase of firing the oscillatory signal may not be able to propagate downstream—due to distractive interference. Here we investigate how synaptic plasticity can facilitate the transmission of oscillatory signal downstream along the information processing pathway in the brain. We show that basic synaptic plasticity rules, that have been reported empirically, are sufficient to generate the required tuning that enables the propagation of the oscillatory signal. In addition, our work presents a synaptic learning process that does not converge to a stationary state, but rather remains dynamic. We demonstrate how the functionality of the system, i.e., transmission of oscillatory activity, can be maintained in the face of constant remodeling of synaptic weights.

## Introduction

It is generally believed that synaptic plasticity is the basis for learning and memory. According to Hebb's rule [1], which is considered the foundation for current views on learning and memory, the interaction strength between two neurons that are co-activated potentiates. This rule has been extended to the temporal domain by taking into account the effect of the causal relation of pre- and post-synaptic firing on the potentiation and depression of the synapse, which is known as spike-timing dependent plasticity (STDP). STDP has been identified in numerous systems in the brain, and a rich repertoire of causal relations has been described [2–12].

Considerable theoretical efforts have been devoted to investigating the possible computational implications of STDP [13–29]. STDP can be thought of as a process of unsupervised learning (for other views such as reward modulated STDP see [30], for example). It has been shown that certain STDP rules can give rise to the emergence of response selectivity at the level of the post-synaptic neuron [14, 15, 20], whereas other STDP rules can provide a homeostatic mechanism that balances the excitatory and inhibitory inputs to the cell [25, 29]. Furthermore, STDP in combination with other plasticity rules has been shown to lead to structure formation in networks [31, 32].

Although 'spike-timing' is explicitly emphasized by the term STDP, most theoretical studies have focused on very basic temporal structures of neuronal activity. Many studies, for example, assume that neuronal firing follows homogeneous Poisson process statistics and that the correlations are instantaneous in time (with a possible small time shift to reflect delayed reactions). However, neuronal oscillatory activity has been reported in such cognitive processes as the encoding of external stimuli, attention, learning and consolidation of memory [33]. Thus, although the specific role of these oscillations in the learning process remains to be determined, it is clear that neuronal oscillations are abundant in the central nervous system. This raises the question of the mechanisms that generate these oscillations: are they genetically hard-wired into the system or can they be acquired via a learning process?

The effect and possible computational role of oscillations on STDP has been addressed in several studies [34–42]. However, in all of these studies the oscillatory activity was either an inherent property of the neuron or inherited via feed-forward connections from inputs that were oscillating and had a clear preferred phase. A recent numerical study indicated that oscillations may emerge in a large scale detailed thalamocortical model with STDP [43]. Nevertheless, it remains unclear whether STDP can give rise to the emergence of oscillatory activity by itself, and if so, under what conditions.

This paper is organized as follows. First, we define the STDP rule and the architecture of the feed-forward model network. Next, we examine the learning dynamics of a single plastic synapse onto a post-synaptic cell, for the case where both pre- and post-synaptic neurons are oscillating. We then investigate the emergence of oscillations in a post-synaptic cell as a result of STDP in a population of feed-forward inputs each of which are oscillating, albeit in different phases such that their net contribution has no defined oscillatory behavior. In this case, if the synaptic weights are uniform or random then the total input to the post-synaptic neuron will have none or very little oscillatory component. We analyze the stability of the homogeneous solution, and show that when the homogeneous solution is not stable the post- synaptic neuron begins to oscillate. However, the synaptic weights themselves do not converge to a fixed point, but rather to a *limit cycle* solution. Finally, we discuss our results and suggest an intuitive explanation for the limit cycle solution.

## Results

### The STDP rule

**Fig 1:**
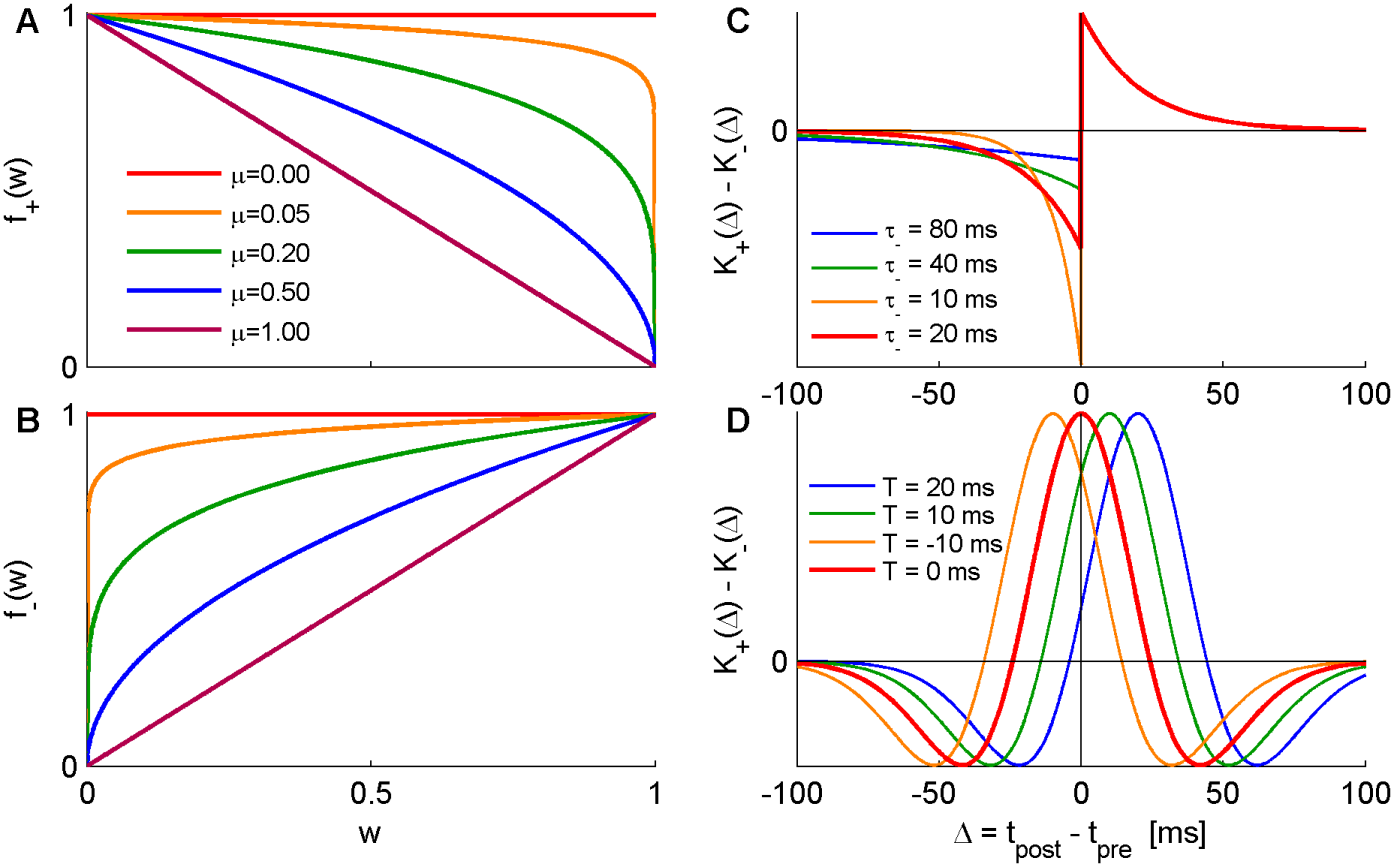
Illustrations of the STDP rules studied in this paper. (A,B) The weight dependence of the STDP rule, *f*_+_(*w*), eq (2) and *f*_−_(*w*), eq (3), with *α* = 1. Shown for different values of *μ* in different colors. (C) The “temporally asymmetric exponential” temporal kernel, eq (4), with *H* = 1 (for Hebbian rule), *τ*_+_ = 20*ms*. Different values of *τ*_−_ are shown in different colors. (D) The “difference of Gaussians” temporal kernel, eq (5), with *τ*_+_ = 20*ms*, *τ*_−_ = 30*ms*, different values of *T*_+_ = T_−_: = *T*shown in different colors.

For convenience, we adopt the STDP formalism presented in [14, 25, 26]. Specifically, we explore the following STDP rule

$$\Delta w=\lambda \left[f_{+}\left(w\right)K_{+}\left(\Delta t\right)-f_{-}\left(w\right)K_{-}\left(\Delta t\right)\right],$$ (1)

where Δ*w* denotes the modification of the weight *w* of a synapse connecting a pre- and post- synaptic neurons, following either a pre- or post- synaptic spike, *λ* is the learning rate, and Δ*t* = *t*_*post*_ −*t*_*pre*_ is the time difference between the pre- and post- synaptic spikes. For simplicity, we assume that all pre-post spike time pairs contribute additively to synaptic plasticity, where the contribution of each such pair follows eq (1). The STDP rule is written as the sum of two processes: potentiation (+) and depression (-). We further assume a separation of variables, such that potentiation and depression are given as the product of the synaptic weight dependence *f*_±_(*w*) and the temporal kernel *K*_±_(Δ*t*) of the STDP rule. Specifically, for the synaptic weight dependence we use the Gütig et al. [14] model

$$f_{+}\left(w\right)={\left(1-w\right)}^{\mu }$$ (2)

$$f_{-}\left(w\right)=\alpha w^{\mu },$$ (3)

where *α* characterizes the relative strength of depression and *μ* the non-linearity of the learning rule (see Fig 1A and 1B). Here, we focused on two families of temporal kernels for the STDP rule. One is a temporally asymmetric exponential rule of the form

$$K_{\pm }(\Delta t)=\frac{e^{\mp H\Delta t/\tau_{\pm }}}{\tau_{\pm }}\Theta \left(\pm H\Delta t\right),$$ (4)

where Θ(*x*) is the Heaviside step function, and *τ*_±_ denote the characteristic timescales of the LTP and LTD branches of the rule, respectively. The parameter *H* allows to controls the nature of the learning rule, with *H* = 1 for a “Hebbian” rule, as in Fig 1C (i.e., potentiating at the causal branch, that is for Δ*t* > 0 when the post fires after pre), and *H* = −1 for an “Anti-Hebbian” STDP rule, see e.g., [12, 44].

The second family of STDP rules considered here uses a difference of Gaussians of the form

$$K_{\pm }(\Delta t)=\frac{1}{\tau_{\pm }\sqrt{2\pi }}e^{-\frac{1}{2}{\left(\frac{\Delta t-T_{\pm }}{\tau_{\pm }}\right)}^{2}}$$ (5)

as temporal kernels, where *τ*_±_, and *T*_±_ are the temporal widths and temporal shifts of the rule respectively, see Fig 1D.

### Single synapse STDP

We first analyze STDP-driven dynamics of a single synapse. We assume that both pre- and post- synaptic neurons are oscillating at the same frequency *ν* albeit with a possible different phase *φ* = *φ*_*pre*_ − *φ*_*post*_. This is done in the limit of weak coupling, assuming that the modification of a single synapse has a marginal effect on the post-synaptic neuron activity. We assume that the pre and post neuronal spiking activity can be described by an inhomogeneous Poisson process statistics with intensity parameter representing instantaneous expected firing rate

$$\langle \rho_{pre/post}\left(t\right)\rangle :=r_{pre/post}\left(t\right)=D_{pre/post}+A_{pre/post}cos\left(\nu t-\varphi_{pre/post}\right),$$ (6)

where $\rho_{pre/post}\left(t\right)=\sum_{i}\delta \left(t-t_{i}^{pre/post}\right)$ denotes the spike trains of the pre/post synaptic cell, as represented by a linear combination of delta functions at the neuron's spike times, with ${\left\{t_{i}^{pre/post}\right\}}_{i=1}^{\infty }$ being the spike times; *r*_pre/post_(*t*) is the instantaneous firing rate of the pre/post- synaptic cell; *D*_pre/post_, *A*_pre/post_ are the mean (DC) and amplitude modulation (AC) of the firing rate, respectively, and *φ*_pre/post_ is the phase of the pre/post- synaptic neuron. The angular brackets 〈⋯〉 denote ensemble averaging.

The pre-post correlation structure is an important factor driving the synaptic dynamics. In the limit of weak coupling, the pre and post firing can be modeled as independent Poisson processes. Consequently, one obtains the following pre-post correlation structure

$$\Gamma \left(\Delta \right):=\frac{\nu }{2\pi }\int_{0}^{\frac{2\pi }{\nu }}\langle \rho_{pre}\left(t\right)\rho_{post}\left(\Delta +\text{t}\right)\rangle dt=\Gamma_{0}\left(1+\Gamma_{r}\text{cos}\left(\nu \Delta +\varphi \right)\right),$$ (7)

where

$$\begin{array}{l} \Gamma_{0}=D_{pre}D_{post},\\ \Gamma_{r}=\left(A_{pre}/D_{pre}\right)\left(A_{post}/D_{post}\right)/2. \end{array}$$ (8)

Note that Γ_*r*_ ≤ 1/2 since the intensity parameter of a Poisson process must be non-negative (*A*_*x*_ ≤ *D*_*x*_).

#### The fixed point of the learning dynamics

In the limit of slow learning dynamics, *λ*→0, we obtain the “mean field” Fokker–Planck approximation for the synaptic dynamics (see e.g., [26] for more details)

$$\frac{1}{\lambda }\frac{d}{dt}w=f_{+}\left(w\right)\int_{-\infty }^{\infty }\Gamma \left(\Delta \right)K_{+}\left(\Delta \right)d\Delta -f_{-}\left(w\right)\int_{-\infty }^{\infty }\Gamma \left(\Delta \right)K_{-}\left(\Delta \right)d\Delta .$$ (9)

Using the pre-post correlation structure of eq (7) yields

$$\frac{1}{\lambda }\frac{d}{dt}w=f_{+}\left(w\right)\Gamma_{0}\left({\bar{K}}_{+}+\Gamma_{r}{\tilde{K}}_{+}^{\nu }\text{cos}\left(\Omega_{+}^{\nu }-\varphi \right)\right)-f_{-}\left(w\right)\Gamma_{0}\left({\bar{K}}_{-}+\Gamma_{r}{\tilde{K}}_{-}^{\nu }\text{cos}\left(\Omega_{-}^{\nu }-\varphi \right)\right),$$ (10)

where

$$\begin{array}{l} {\bar{K}}_{\pm }:=\int_{-\infty }^{\infty }K_{\pm }\left(\Delta \right)d\Delta ,\\ {\tilde{K}}_{\pm }^{\nu }e^{i\Omega_{\pm }^{\nu }}:=\int_{-\infty }^{\infty }e^{-i\nu \Delta }K_{\pm }\left(\Delta \right)d\Delta . \end{array}$$ (11)

Where ${\tilde{K}}_{\pm }^{\nu }$ are the absolute values of the Fourier transforms, and $\Omega_{\pm }^{\nu }$ are real. Note that our kernel functions are normalized (eqs (4) and (5)), such that ${\bar{K}}_{\pm }=1$ by construction.

The fixed point of the synaptic dynamics ${\frac{dw}{dt}|}_{w^{*}}=0$ obeys

$$\frac{f_{+}}{f_{-}}=\frac{1}{\alpha }{\left(\frac{1-w^{*}}{w^{*}}\right)}^{\mu }=\frac{{\bar{K}}_{-}+\Gamma_{r}{\tilde{K}}_{-}^{\nu }\text{cos}\left(\Omega_{-}^{\nu }-\varphi \right)}{{\bar{K}}_{+}+\Gamma_{r}{\tilde{K}}_{+}^{\nu }\text{cos}\left(\Omega_{+}^{\nu }-\varphi \right)}\equiv Q\left(\varphi \right).$$ (12)

The solution of the fixed point equation yields

$$w^{*}\left(\varphi \right)=\frac{1}{{\left(\alpha Q\left(\varphi \right)\right)}^{1/\mu }+1},$$ (13)

where the notation w*(*φ*) underscores the dependence of the fixed point solution on the relative phase of the pre and post neuronal mean activity. Additionally, eq (13) shows the different effects of the parameters that govern the STDP-driven synaptic dynamics on the fixed point solution.

**Fig 2:**
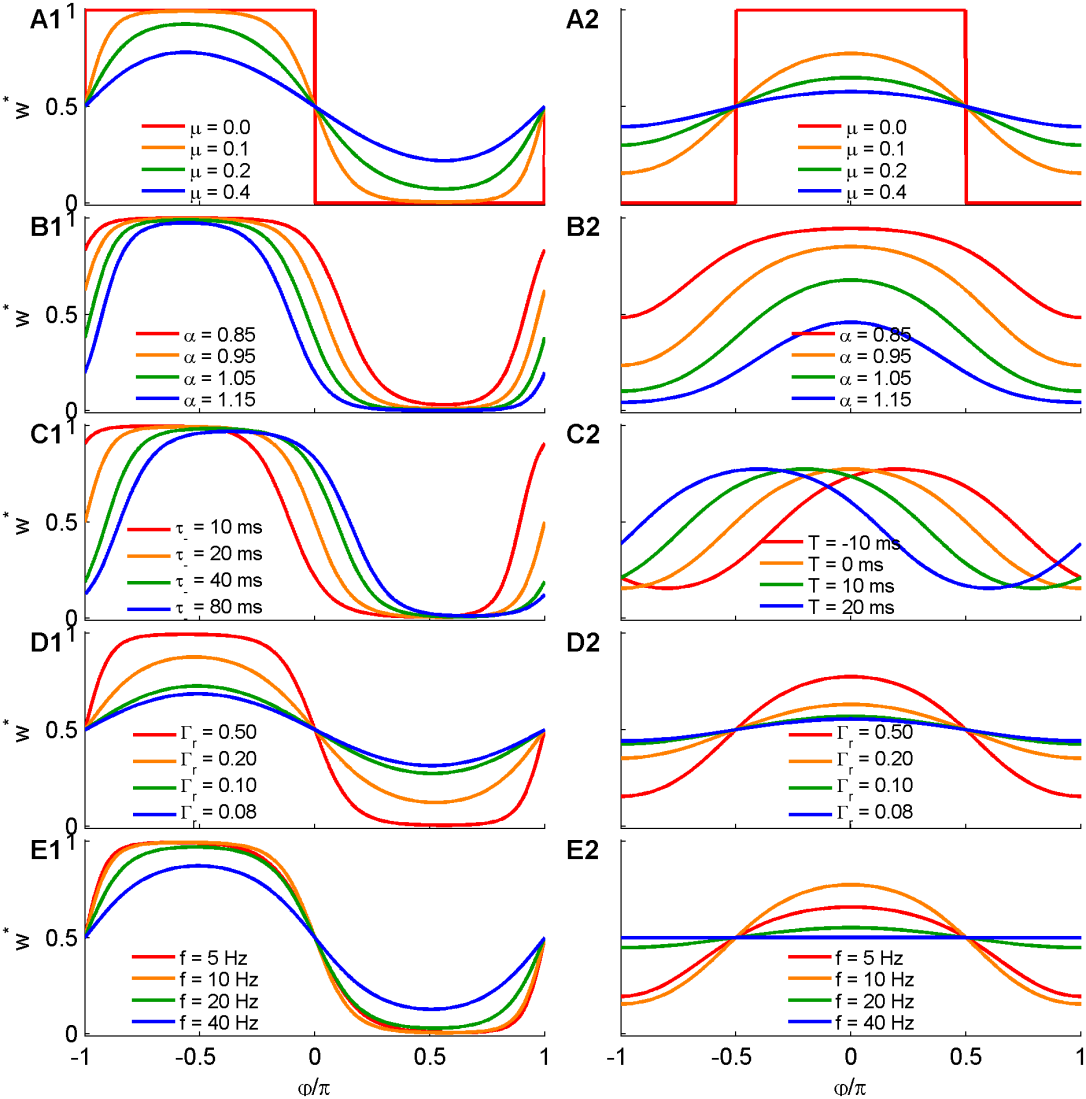
The asymptotic synaptic weight in the case of single synapse STDP. The different panels show the resultant synaptic weight profile w*(*φ*) given by eqs (12) and (13) as a function of the pre-post phase difference, *φ*, for different sets of parameters. A1-E1 (left column) show the results for the exponential STDP kernel, as in Fig 1C. A2-E2 (right column) show the results for the difference of Gaussians STDP kernel, as in Fig 1D. The different colors show the variation of the asymptotic weight with respect to a single parameter of the STDP-driven synaptic dynamics that is being varied. In A1 and A2 the different colors depict different values of *μ*. In B1 and B2 the different colors depict different values of the relative strength of depression *α*. In C1 and C2 the different colors are for different time constants of the STDP rule *τ*_−_ and *T*, respectively. Panels D1 and D2 show the effect of the relative strength of the oscillatory activity Γ_*r*_, see eq (8). Panels E1 and E2 show the effect of the oscillation frequency *f*. In all panels, unless stated otherwise in the legend the following parameters were used: *α* = 1, *μ* = 0.1, *ν* = 2*π* · 10*Hz*, and Γ_*r*_ = 0.5, for the exponential STDP kernel (left column) *τ*_+_ = *τ*_−_ = 20*ms* was used, and for the Gaussian STDP kernel (right column) *T*_±_ = 0 and *τ*_+_ = 20*ms*, *τ*_−_ = 30*ms* were used.

Fig 2A1 shows w*(*φ*) for different values of *μ* (depicted in different colors) for the temporally asymmetric Hebbian (exponential) rule with *τ*_+_ = *τ*_−_. As can be seen from the figure, this rule favors negative phases, −*π* < *φ* = *φ*_*pre*_ − *φ*_*post*_ < 0. In the additive case (i.e., for *μ* = 0) w*(*φ*) is a step function with a discontinuity at *φ* ∈ {0, *π*}. Increasing *μ* results in a smoother profile that does not alter the width of the profile. The width of the profile is governed by the relative strength of the depression, *α*. Decreasing *α* widens the profile (consider for example the width at half height or region in which the synaptic weights are larger than 0.5), whereas increasing *α* narrows it, Fig 2B1.

The structure of the profile, and in particular the ‘preferred phase’, is governed by the temporal kernel of the STDP rule. Whereas the temporally asymmetric Hebbian rule prefers negative phase differences (causal), the temporally symmetric rule prefers phase differences that are small in their absolute value, Fig 2A1 and 2A2.

To quantify the width of the profile, it is convenient to use the *μ*-invariant solution that results from the fact that for w*(*φ*_*c*_) = 1/2 the ratio *f*_+_/*f*_−_ is independent of *μ*. From eq (13), the *μ*-invariant solution *φ*_*c*_ is determined by

$$\alpha {\tilde{K}}_{-}^{\nu }\text{cos}\left(\Omega_{-}^{\nu }-\varphi_{c}\right)-{\tilde{K}}_{+}^{\nu }\text{cos}\left(\Omega_{+}^{\nu }-\varphi_{c}\right)=\left(1-\alpha \right)/\Gamma_{r}.$$ (14)

Thus *φ*_*c*_ yields the phases in which the profile crosses w* = 1/2; hence, they can be used to characterize the “profile width”.

In the temporally asymmetric exponential rule one obtains

$$\begin{array}{cc} {\tilde{K}}_{\pm }^{\nu }={\left(\nu^{2}\tau_{\pm }{}^{2}+1\right)}^{-1/2}, & \Omega_{\pm }^{\nu }=\mp H\text{arctan}\left(\nu \tau_{\pm }\right) \end{array}.$$ (15)

For this rule, with *α* = 1 one obtains $\varphi_{c}=\text{arctan}\left(\frac{{\left(\nu \tau_{-}\right)}^{2}-{\left(\nu \tau_{+}\right)}^{2}}{\nu \tau_{-}[1+{\left(\nu \tau_{+}\right)}^{2}]+\nu \tau_{+}[1+{\left(\nu \tau_{-}\right)}^{2}]}\right)$. Thus, for *τ*_+_ = *τ*_−_ the solutions are *φ*_*c*_ = 0,−*π*. As the ratio *τ*_+_ = *τ*_−_ is increased, both solutions are increased, and the profile rotates clockwise on the ring, but its width remains *π*, Fig 2C1.

For the Gaussian kernels one obtains

$$\begin{array}{cc} {\tilde{K}}_{\pm }^{\nu }=e^{-\frac{1}{2}{\left(\nu \tau_{\pm }\right)}^{2}}, & \Omega_{\pm }^{\nu }=-\nu T_{\pm } \end{array}.$$ (16)

In the case of a temporally shifted “Mexican hat” rule; i.e., *T*_+_ = *T*_−_: = *T*, the *μ*-invariant solutions are symmetric around *νT*: *φ*_*c*_ = −*νT* ± *β*, with the half width $\beta =\text{arccos}\left(\frac{\alpha -1}{\Gamma_{r}(K_{+}-\alpha K_{-})}\right)$, Fig 2C2. For this rule, *α* = 1 implies a half width of *β* = *π*/2.

From eq (14), for *α* = 1, the parameters that characterize the mean firing rate (DC) and modulation amplitude (AC) do not affect the profile width. However, the AC to DC ratio, Γ*r*, will affect the circular variance of the synaptic weight profile for *μ* ≠ 0 (see eq (12)), Fig 2D1 and 2D2.

The Fourier transforms of the STDP rule ${\tilde{K}}_{\pm }^{\nu }$ are monotonically decreasing functions of the oscillation frequency *ν*. Increasing the frequency decreases ${\tilde{K}}_{\pm }^{\nu }$ and causes *Q*(*φ*) to converge towards 1. Hence, in the high frequency limit the profile becomes independent of the phase and converges to $w^{*}\left(\varphi \right)=\frac{1}{\alpha^{1/\mu }+1}$, see Fig 2E1 and 2E2. For both types of temporal kernels studied here, the characteristic frequency for the decay is 1/*τ*. Thus, the synaptic dynamics loses its sensitivity to the oscillations when the relevant timescale of the STDP rule is on the order of the period of the oscillations. Note however that due to the discontinuity in the STDP profile of the exponential rule the Fourier transform of its temporal kernel decays algebraically fast (i.e., ${\tilde{K}}_{}^{\nu }\propto \nu^{-1}$ for large *ν*) with the oscillation frequency *ν*, whereas the Fourier transform of the smooth Gaussian model decays exponentially fast with *ν* (i.e., ${\tilde{K}}_{}^{\nu }=e^{-{\left(\nu \tau \right)}^{2}/2}$ for large *ν*).

### Excitatory synapse population

**Fig 3:**
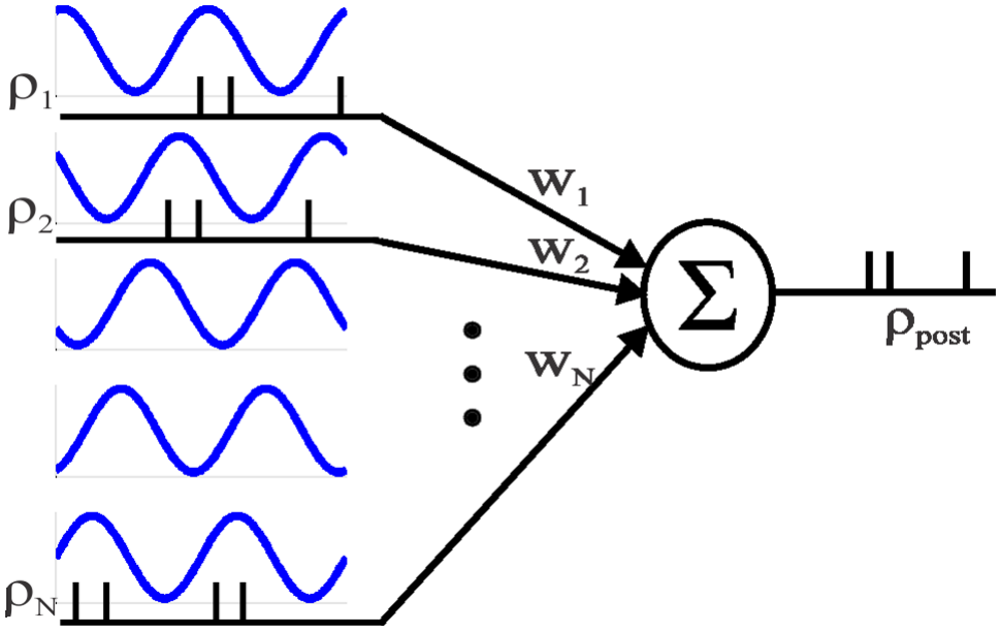
A schematic description of the network architecture showing the pre-synaptic population of oscillating neurons serving as feed-forward input to a single post-synaptic neuron.

We now want to consider in which ways oscillatory activity affects the STDP-driven dynamics of a synaptic population of *N* excitatory neurons that project onto a single post-synaptic neuron in a feed-forward manner, see Fig 3. All the excitatory synapses obey the same STDP rule.

#### The isotropic “Ring Model”

The spiking activity of the pre-synaptic population ${\left\{\rho_{j}\left(t\right)\right\}}_{j=1}^{N}$ is modeled by an independent inhomogeneous Poisson processes with instantaneous mean firing rates that are oscillating in time. We further assume that all pre-synaptic neurons are oscillating at the same angular frequency *ν*, same mean firing rate *D*, and same rate modulation *A*, albeit with phases that are evenly spaced on the ring

$$\;\begin{array}{cc} \langle \rho_{j}\left(t\right)\rangle =D+Acos\left(\nu t-\varphi_{j}\right), & \varphi_{j}=2\pi j/N \end{array},$$ (17)

where 〈*ρ*_*j*_(*t*)〉 is the mean firing rate of the *j*’th pre-synaptic neuron. Note that in this model the net activity of the pre-synaptic population $\sum_{j}\langle \rho_{j}\left(t\right)\rangle$ is constant in time. Using the independent Poisson process statistics yields

$$\begin{array}{l} \Gamma_{jk}\left(\Delta \right):=\frac{\nu }{2\pi }\int_{0}^{\frac{2\pi }{\nu }}\langle \rho_{j}\left(t\right)\rho_{k}\left(t+\Delta \right)\rangle dt,\\ \;=D^{2}+\frac{A^{2}}{2}\text{cos}\left(\nu \Delta +\left(\varphi_{j}-\varphi_{k}\right)\right)+\delta_{jk}\delta \left(\Delta \right)D. \end{array}$$ (18)

#### The post-synaptic neuron model

We model the post-synaptic activity by a “delayed linear Poisson neuron”, which has been frequently used in studies on STDP, see e.g. [14, 18, 19, 25, 45]. The post-synaptic activity obeys a Poisson process statistics with a mean instantaneous firing rate that is given by

$$\langle \rho_{post}\left(t\right)\rangle =\frac{1}{N}\sum_{j=1}^{N}w_{j}\rho_{j}\left(t-d\right),$$ (19)

where d>0 is a characteristic delay of the post-synaptic neuron response, and *w*_*j*_ is the weight of the *j*’th synapse. The synaptic dynamics is driven by the overlap of the pre-post correlations and the STDP rule. In the linear Poisson model, eq (19), the pre-post correlations are determined by the pairwise correlation structure of the input layer, and the synaptic weights

$$\begin{array}{l} \Gamma_{j,\text{post}}\left(\Delta \right)=\frac{1}{N}\sum_{k=1}^{N}w_{k}\Gamma_{jk}\left(\Delta -d\right)\\ =\frac{D}{N}\delta \left(\Delta -d\right)w_{j}+\frac{D^{2}}{N}\overset{N}{\underset{k=1}{\sum }}w_{k}+\frac{A^{2}}{2N}\overset{N}{\underset{k=1}{\sum }}w_{k}\text{cos}\left(\nu \left(\Delta -d\right)+\left(\varphi_{j}-\varphi_{k}\right)\right). \end{array}$$ (20)

For large *N* it is convenient to take the continuum limit; hence replacing the discrete sum over the phases ${\left\{\varphi_{j}\right\}}_{j=1}^{N}$ by integration over *φ*, and replacing *w*_*j*_ by the function *w*(*φ*):

$$\Gamma \left(\varphi ,\Delta \right)=\frac{D}{N}\delta \left(\Delta -d\right)w\left(\varphi \right)+D^{2}\bar{w}+\frac{A^{2}}{2}\text{cos}\left(\varphi +\nu \left(\Delta -d\right)-\psi \right)\tilde{w},$$ (21)

where $\bar{w}$ and $\tilde{w}e^{i\psi }$ are order parameters that describe the synaptic weight distribution,

$$\begin{array}{l} \bar{w}:=\int_{-\pi }^{\pi }w\left(\phi \right)\frac{d\phi }{2\pi },\\ \tilde{w}e^{i\psi }:=\int_{-\pi }^{\pi }e^{i\phi }w\left(\phi \right)\frac{d\phi }{2\pi }. \end{array}$$ (22)

Thus, $\bar{w}$ is the mean (DC component) of the synaptic weight profile *w*(*φ*) and $\tilde{w}e^{i\psi }$ its first Fourier component. The phase *ψ* is determined by the condition that $\tilde{w}$ is real and non-negative. We also refer to *ψ* intuitively as the “center of mass” of the weight profile.

#### The mean field Fokker-Planck equations

In the limit of a slow learning rate, *λ*→0, one obtains (see e.g., [26] for a detailed derivation of the mean field equations)

$$\dot{w}\left(\varphi ,t\right)/\lambda =f_{+}\left(w\left(\varphi ,t\right)\right)\int_{-\infty }^{\infty }\Gamma \left(\varphi ,\Delta \right)K_{+}\left(\Delta \right)d\Delta -f_{-}\left(w\left(\varphi ,t\right)\right)\int_{-\infty }^{\infty }\Gamma \left(\varphi ,\Delta \right)K_{-}\left(\Delta \right)d\Delta .$$ (23)

Using the spatiotemporal correlation structure induced by the oscillations, eq (21), we obtain

$$\dot{w}\left(\varphi ,t\right)/\lambda =\bar{w}\left(t\right)F_{0}\left(\varphi ,t\right)+\tilde{w}\left(t\right)F_{1}\left(\varphi ,t\right),$$ (24)

where we neglected the first term of eq (21) which is *O*(1/N) in the limit of large *N*. The terms *F*_0_ and *F*_1_ are given by

$$\begin{array}{l} F_{0}\left(\varphi ,t\right):=D^{2}\left({\bar{K}}_{+}f_{+}\left(w\left(\varphi ,t\right)\right)-{\bar{K}}_{-}f_{-}\left(w\left(\varphi ,t\right)\right)\right),\\ F_{1}\left(\varphi ,t\right):=\frac{A^{2}}{2}\{{\tilde{K}}_{+}^{\nu }f_{+}\left(w\left(\varphi ,t\right)\right)\text{cos}\left(\varphi -\Omega_{+}^{\nu }-\nu d-\psi \left(t\right)\right)\\ \;-{\tilde{K}}_{-}^{\nu }f_{-}\left(w\left(\varphi ,t\right)\right)\text{cos}\left(\varphi -\Omega_{-}^{\nu }-\nu d-\psi \left(t\right)\right)\}. \end{array}$$ (25)

Integrating eq (24) over *φ* (see e.g., Ben Yishai et al. [46] for details) we obtain the dynamics of the order parameters $\bar{w}$, $\tilde{w}$, and *ψ*:

$$\frac{1}{\lambda }\frac{d}{dt}\bar{w}(t)=\bar{w}\left(t\right){\bar{F}}_{0}\left(t\right)+\tilde{w}\left(t\right){\bar{F}}_{1}\left(t\right),$$ (26)

$$\begin{array}{cc} \begin{array}{l} \frac{1}{\lambda }\frac{d}{dt}\left(\tilde{w}\left(t\right)e^{i\psi \left(t\right)}\right) \end{array} & \begin{array}{l} :=\frac{e^{i\psi \left(t\right)}}{\lambda }\left(\frac{d}{dt}\tilde{w}\left(t\right)+i\tilde{w}\left(t\right)\frac{d}{dt}\psi \left(t\right)\right)\\ =\bar{w}\left(t\right){\tilde{F}}_{0}\left(t\right)e^{i\Phi_{0}\left(t\right)}+\tilde{w}\left(t\right){\tilde{F}}_{1}\left(t\right)e^{i\Phi_{1}\left(t\right)}, \end{array} \end{array}$$ (27)

where ${\bar{F}}_{x}\left(t\right):=\int_{-\pi }^{\pi }F_{x}\left(\phi ,t\right)\frac{d\phi }{2\pi }$ and ${\tilde{F}}_{x}\left(t\right)e^{i\Phi_{x}\left(t\right)}:=\int_{-\pi }^{\pi }e^{i\phi }F_{x}\left(\phi ,t\right)\frac{d\phi }{2\pi }$ (*x* = 0, 1).

#### The homogeneous synaptic steady state

Due to the symmetry of the mean field eq (23) with respect to the phase *φ*, a uniform solution *w*(*φ*,*t*) = *w*_*h*_ always exists. In this case $\tilde{w}\left(t\right)=0$, and the steady state equation reduces to

$$0=D^{2}w_{h}\left({\bar{K}}_{+}f_{+}\left(w_{h}\right)-{\bar{K}}_{-}f_{-}\left(w_{h}\right)\right).$$ (28)

The trivial solution *w*_*h*_ = 0 is of no biological interest. In our definitions we used ${\bar{K}}_{+}={\bar{K}}_{-}:=\bar{K}$, consequently a non-trivial homogeneous solution to eq (28) for *μ* > 0 obeys *f*_+_(*w*_*h*_) = *f*_−_(*w*_*h*_), yielding

$$w_{h}=\frac{1}{1+\alpha^{1/\mu }}.$$ (29)

For such a uniform profile, the post-synaptic activity as given by eq (19) becomes: $\langle \rho_{post}\left(t\right)\rangle =\frac{w_{h}}{N}\sum_{j=1}^{N}\left[D+Acos\left(\nu \left(t-d\right)-\varphi_{j}\right)\right]=Dw_{h}$. Consequently, the post-synaptic cell will not oscillate in the homogeneous case.

#### Stability of the homogeneous steady state

To study the stability of the homogeneous solution, we consider an arbitrary (though small) fluctuation *w*(*φ*_*j*_) = *w*_*h*_ + *δw*(*φ*_*j*_) and expand the synaptic dynamics around the homogeneous fixed point to a leading order in the fluctuations: $\delta \dot{w}=\lambda M\delta w$. The stability of the fixed point is determined by the eigenvalues of the stability matrix **M**. Due to the symmetry of the problem in the given case, it is sufficient to study the stability with respect to fluctuations in the uniform direction $\bar{w}$ and in the direction of the first Fourier mode $\tilde{w}$, obtaining: $\delta \dot{\bar{w}}=\lambda m_{0}\delta \bar{w}$ and $\delta \dot{\tilde{w}}=\lambda m_{1}\delta \tilde{w}$; thus, the uniform and the first Fourier mode are eigenvectors of the stability matrix, with *m*_0_ and *m*_1_ as their corresponding eigenvalues. We obtain

$$m_{0}=w_{h}{\frac{\partial F_{0}}{\partial w}|}_{w_{h}}$$ (30)

and

$$m_{1}=\frac{1}{2}m_{0}+{\tilde{F}}_{1}^{}(w_{h}).$$ (31)

At the homogeneous fixed point (which obeys *f*_+_(*w*_*h*_) = *f*_−_(*w*_*h*_) = (1−*w*_*h*_)^*μ*^) one obtains $m_{0}=-D^{2}\bar{K}{\left(1-w_{h}\right)}^{\mu }\frac{\mu }{1-w_{h}}$. Thus, for *μ*>0, the homogeneous fixed point is always stable with respect to fluctuations in the uniform direction (see also [14]).

In contrast, the homogeneous fixed point is not always stable with respect to fluctuations in the direction of $\tilde{w}$. Namely,

$$m_{1}=\frac{1}{2}D^{2}\bar{K}{\left(1-w_{h}\right)}^{\mu }\left[\frac{-\mu }{1-w_{h}}+\frac{A^{2}}{2D^{2}}\left(\frac{{\tilde{K}}_{+}^{\nu }}{\bar{K}}\text{cos}\left(\Omega_{+}^{\nu }+\nu d\right)-\frac{{\tilde{K}}_{-}^{\nu }}{\bar{K}}\text{cos}\left(\Omega_{-}^{\nu }+\nu d\right)\right)\right].$$ (32)

The first Fourier eigenvalue *m*_1_ is a sum of two terms. The first term is a stabilizing term *m*_0_/2 that is linear in *μ* in the limit of small *μ* (for *α*≥1, note that for *α>*1, *w*_*h*_→0 in the limit of small *μ*, and for *α* = 1, *w*_*h*_ = 1/2 for all *μ*). The second term scales with (*A*/*D*)^2^, and its sign is determined by the sign of ${\tilde{K}}_{+}^{\nu }\text{cos}(\Omega_{+}^{\nu }+\nu d)-{\tilde{K}}_{-}^{\nu }\text{cos}(\Omega_{-}^{\nu }+\nu d)$. Thus, for *α*≥1, if $\left({\tilde{K}}_{+}^{\nu }\text{cos}(\Omega_{+}^{\nu }+\nu d)-{\tilde{K}}_{-}^{\nu }\text{cos}(\Omega_{-}^{\nu }+\nu d)\right)>0$, there exists a critical value of the non-linearity parameter *μ*_*c*_, such that the homogenous fixed point loses its stability for *μ<μ*_*c*_. As *w*_*h*_≤1/2 for *α*≥1 (applicable in the excitatory case—compare with Gütig et al. [14]) *μ*_*c*_ satisfies:

$$\mu_{c}\geq \frac{A^{2}}{4D^{2}}\left(\frac{{\tilde{K}}_{+}^{\nu }}{\bar{K}}\text{cos}\left(\Omega_{+}^{\nu }+\nu d\right)-\frac{{\tilde{K}}_{-}^{\nu }}{\bar{K}}\text{cos}\left(\Omega_{-}^{\nu }+\nu d\right)\right).$$ (33)

Thus, for example, in the case of a “Mexican hat” type STDP rule, which is a temporally symmetric difference of Gaussians kernel with *T*_+_ = *T*_−_ = 0, and *τ*_+_ < *τ*_−_ (see eq (5)), one obtains $\begin{array}{cc} {\tilde{K}}_{\pm }^{\nu }=e^{-\frac{1}{2}{\left(\nu \tau_{\pm }\right)}^{2}}, & \Omega_{\pm }^{\nu }=0 \end{array}$, (see eq (16)). Consequently, an oscillation frequency that obeys |*νd*| < *π*/2 yields *μ*_*c*_>0 for any positive value of *A*. In contrast, for this frequency regime, *m*_1_ is negative in the case of an inverted Mexican hat STDP rule; i.e., for *τ*_−_ < *τ*_+_.

In the case of a temporally anti-symmetric STDP rule, that is, the exponential kernel of eq (4) with *τ*_+_ = *τ*_−_: = *τ*, one obtains ${\tilde{K}}_{+}^{\nu }={\tilde{K}}_{-}^{\nu }$ and $\Omega_{-}^{\nu }=-\Omega_{+}^{\nu }=H\text{arctan}(\nu \tau )$, see eq (15). As a result, up to a positive factor the right hand side of eq (33) is *H* sin(*νd*); hence, *μ*_*c*_>0 for *νd* < *π* for the case of a Hebbian rule, *H* = 1, whereas for the anti-Habbian rule, *H* = −1, *m*_1_ is negative for *νd* < *π*, and the homogeneous state is expected to be stable. Note that in the above examples we did not fully describe all the regimes in which *μ*_*c*_ > 0.

#### Numerical simulations

As the uniform solution loses its stability to fluctuations in the direction of $\tilde{w}$ one might naively expect that the synaptic dynamics would converge to another fixed point in which the post-synaptic neuron develops a phase preference, and begins to oscillate.

**Fig 4:**
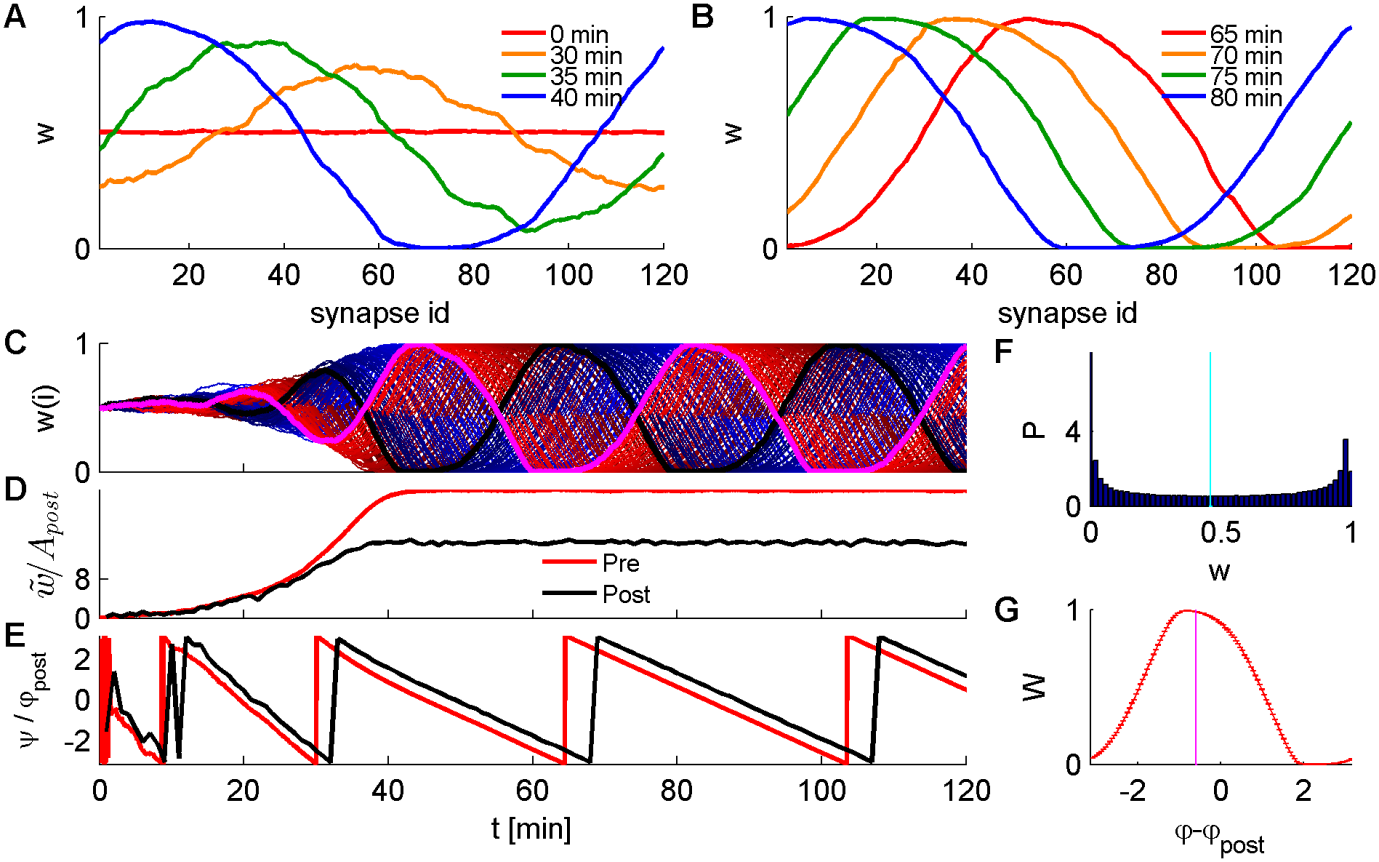
Numerical simulation of STDP-driven synaptic dynamics of a population of 120 excitatory synapses providing feed-forward input onto a single conductance based post-synaptic neuron (see e.g. Fig 3 and Methods for details). The pre-synaptic activity followed an inhomogeneous Poisson process with a time-varying intensity by eq (17), using *N* = 120, *D* = 10*Sp*/*sec*, *A* = 10*Sp*/sec, *ν* = 2*π*·10*Hz*. We simulated the temporally anti-symmetric exponential STDP rule (eq (4) with *τ*_±_ = 20*ms*), with *α* = 1.1, *μ* = 0.1, using a learning rate constant *λ* = 5·10^−4^. (A and B) The synaptic weight profile *w*(*φ*, *t*) as a function of *φ* is shown for different times *t* = 0, 35,…80min by the different colors. The solid lines show the temporal average of *w*(*φ*, *t*) on the interval [*t*,*t*+1min]. (C) Traces showing the dynamics of all 120 synapses, each shown in a different color: black for the 60^th^ synapse (*φ*_60_ = *π*), magenta for 120^th^ synapse (*φ*_120_ = 2*π*), shades of blue for synapses 1–59 and shades of red for synapses 61–119. (D) The dynamics of the order parameter $\tilde{w}$ (eq (22)) shown in red (x100 scaled), and the oscillation amplitude of post-synaptic activity *A*_*post*_ (eq (6)) shown in black and measured in Sp/sec, binned in time-bins of 1 minute. (E) The dynamics of the order parameter *ψ* (eq (22)) shown in red (calculated in time-bins of 1 sec), and the oscillation phase of post-synaptic activity *φ*_*post*_ (calculated in time-bins of 1 min). (F) The distribution of synaptic weights of all 120 synapses, calculated using the second half of the simulation (after convergence to a limit cycle solution). The vertical cyan line denotes the mean value of the distribution. (G) The characteristic shape of the propagating wave, calculated from synaptic weights during the second half of the simulation. Error bars depict the standard error of the mean (calculated from measurements binned at 1sec during the second half of the simulation). The calculation of *φ*_*post*_ was obtained using time-bins of 1 min. The vertical magenta line shows the mean phase difference between pre- and post- synaptic activity.

Fig 4 shows a typical example of a numerical simulation of the synaptic dynamics using a conductance- based post-synaptic neuron (see Methods for details). The initial conditions in the simulations were uniform, *w*(*φ*,*t* = 0) = 1/2, for all *φ*. The uniform solution in this case is not stable. Some synapses increase their weight and some decrease and this process does not appear to converge.

Fig 4A shows the synaptic weight profile at different times. The red line depicts the weight profile averaged over the first minute, and is not identically flat due to the inherent stochasticity of the learning dynamics. Due to such random fluctuations a small preference for a certain phase is generated, which in turn is then amplified by the synaptic dynamics (*m*_1_>0). As a result, after several minutes of STDP-driven dynamics the synaptic weights profile developed a pronounced phase preference. However, this profile was not stable, but rather drifted in time, see Fig 4B.

Fig 4C shows the dynamics of the entire synaptic population that appear to oscillate. However it is more convenient to view the limit cycle solution in terms of the order parameters, see Fig 4D and 4E. As can be seen from the figure, both $\bar{w}$ and $\tilde{w}$ converge after about 60 minutes of simulation time. During this period, the synaptic weight profile develops a unimodal hill shape. The structure of this shape is stable; namely, its mean, width, amplitude modulation, etc. are fixed in time, see Fig 4F and 4G. However, its “center of mass” *ψ* drifts in time with a constant angular velocity, see Fig 4E.

#### The limit cycle solution

To study the propagating wave solution we apply the limit-cycle ansatz

w(φ,t)=W(φ−Vt), (34)

where *W*(*ϕ*) is a steady state profile of the propagating wave, see e.g., Fig 4G, and *V* is its angular velocity. Under the limit cycle ansatz the order parameters obey

$$\begin{array}{ccc} \bar{w}\left(t\right)=\bar{W}, & \tilde{w}\left(t\right)=\tilde{W}, & \psi \left(t\right)=\psi_{0}+Vt \end{array},$$ (35)

and one obtains

$$\begin{array}{cccc} {\bar{F}}_{x}\left(t\right)={\bar{F}}_{x}, & {\tilde{F}}_{x}\left(t\right)={\tilde{F}}_{x}, & \Phi_{x}\left(t\right)=\Phi_{x}+Vt, & x=0,1 \end{array}.$$ (36)

Substituting eqs (35) and (36) into the dynamics of the order parameters, eqs (26) and (27), yields

$$0=\bar{W}{\bar{F}}_{0}+\tilde{W}{\bar{F}}_{1},$$ (37)

$$i\tilde{W}\frac{V}{\lambda }=\bar{W}{\tilde{F}}_{0}e^{i\left(\Phi_{0}-\psi_{0}\right)}+\tilde{W}{\tilde{F}}_{1}e^{i\left(\Phi_{1}-\psi_{0}\right)}.$$ (38)

From eq (38) one obtains that the drift velocity *V* scales linearly with the learning rate *λ*. Although eqs (37) and (38) seem to be a simple set of equations for the order parameters, $\bar{W}$ and $\tilde{W}$, solving them is not trivial. This is because ${\bar{F}}_{0,1}$ and ${\tilde{F}}_{0,1}$ also depend on higher order Fourier modes of the synaptic weight profile. However, in the special case of “zero drift”, *V* = 0, one can obtain a closed form set of equations that only involve the order parameters $\bar{W}$ and $\tilde{W}$.

#### The case of zero drift velocity

The zero drift velocity corresponds to a fixed point solution for the synaptic weights. In this case, the input to the post-synaptic neuron is a weighted sum of inhomogeneous Poisson processes that the intensities of which are each cosine modulated in time. Hence, the total input to the post-synaptic neuron is also cosine modulated. As a result, in the linear Poisson model, the mean rate of the post-synaptic neuron is cosine modulated, conforming to eq (6) with

$$\begin{array}{ccc} D_{post}=D\bar{W}, & A_{post}=A\tilde{W}, & \varphi_{post}=\psi_{0}+\nu d \end{array}.$$ (39)

Consequently, the fixed point (zero drift) solution for *w*(*φ*) is given by eq (13) as

$$Q\left(\varphi \right)=\frac{1+\Gamma_{r}{\tilde{K}}_{-}^{\nu }\text{cos}\left(\varphi -\Omega_{-}^{\nu }-\psi_{0}-\nu d\right)}{1+\Gamma_{r}{\tilde{K}}_{+}^{\nu }\text{cos}\left(\varphi -\Omega_{+}^{\nu }-\psi_{0}-\nu d\right)}.$$ (40)

Note that the approximation of neglecting terms that are *O*(1/*N*) in the transition to eq (24) conforms with the assumption of the “weak coupling limit” in eq (7). Eq (13) needs to be solved self- consistently; i.e., the parameters $\bar{W},\tilde{W},\psi_{0}$ are both determined by *Q*(*φ*) and determine *Q*(*φ*) via $\Gamma_{r}=\frac{A^{2}\tilde{W}}{2D^{2}\bar{W}}$. The self-consistent equations yield conditions on the modulation parameters *D*, *A*, *ν* of eq (17) that yield a zero drift.

#### Solution in the additive rule

**Fig 5:**
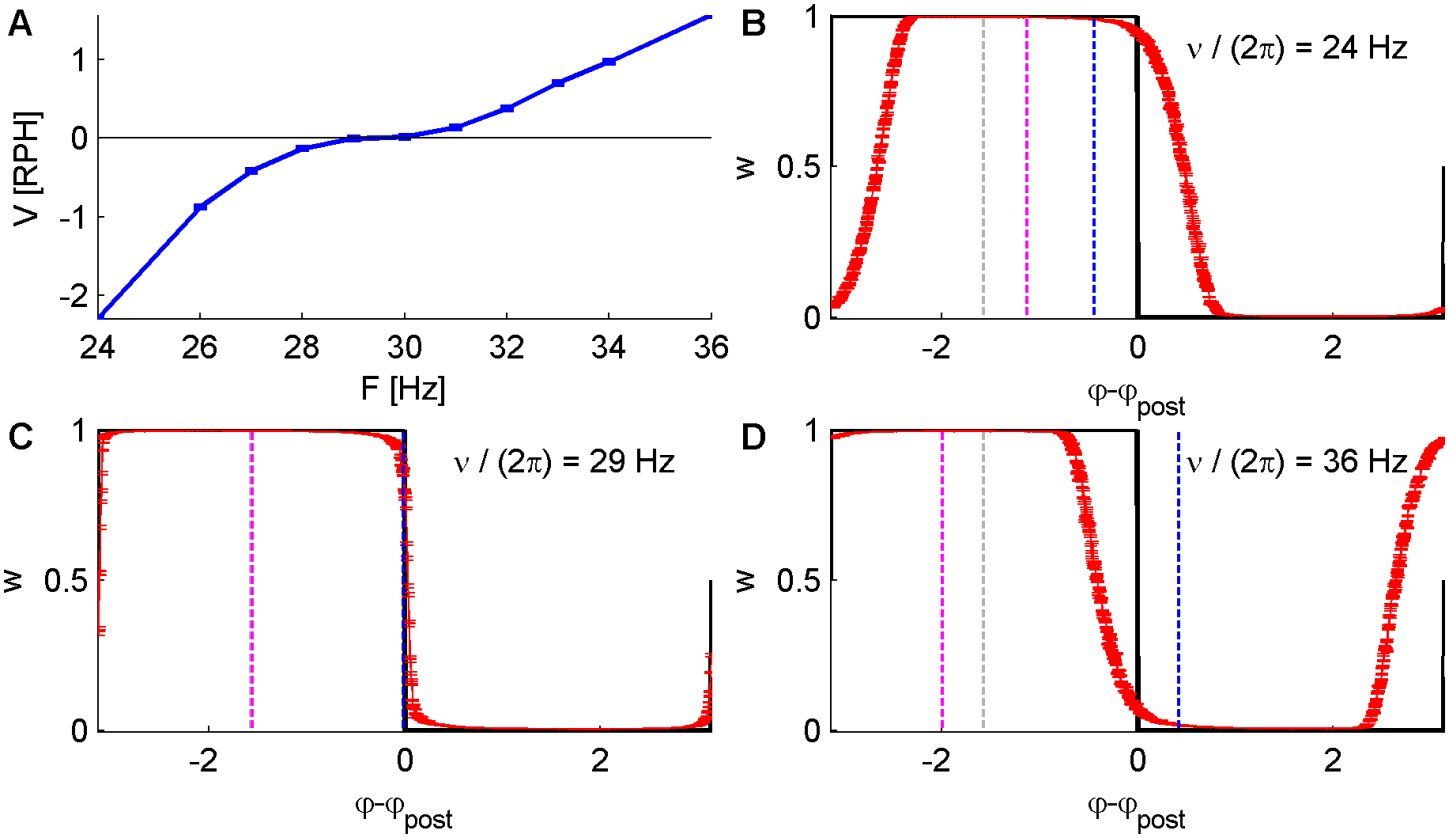
Numerical simulations of the STDP-driven synaptic dynamics of 1200 excitatory synapses in an isotropic “Ring Model” architecture, serving as feed-forward input onto a single conductance based post-synaptic neuron (see Methods for details). The pre-synaptic activity followed eq (17), with *N* = 1200, *D* = 10*Sp*/sec, *A* = 10*Sp*/sec and a varying angular frequency *ν*. Here we simulated the temporally anti-symmetric exponential STDP rule (eq (4) with *τ*_±_ = 20*ms*), with *α* = 1, *μ* = 0, using a learning rate constant *λ* = 5·10^−3^. (A) The angular velocity *V* of the order parameter *ψ* (eq (34) in Revolutions per Hour—RPH) is plotted as a function of the oscillation frequency. (B, C and D) The synaptic weight profile as estimated numerically (calculated from the second half of the simulations, following convergence to the limit cycle), shown as a function of the pre-post phase difference, in red. Note the weight profile and the post phase drift at the same velocity, in red. The black curve shows the zero drift solution of eq (40). The vertical dashed gray line depicts the order parameter or “center of mass” *ψ*_0_ of the zero drift solution, the dashed blue line shows *ψ*_0_ + *νd*, and the pink line shows the “center of mass” *ψ* of the weight profile of the limit cycle solution. The panels differ in terms of the oscillation frequency of the pre-synaptic population $\frac{\nu }{2\pi }=24,\;29$ and 36 Hz for panels B, C, and D, respectively.

For simplicity, we focus on the “additive STDP rule” (i.e.; the case of *μ* = 0) in the following. For the case of the additive rule the synaptic weight profile *w*(*φ*) is binary, see e.g. Fig 2A1 and 2A2 Hence,

$$w\left(\varphi \right)=\{\begin{array}{ll} 1, & \varphi \in (\varphi_{c1},\varphi_{c2})\\ 0, & \text{otherwise} \end{array},$$ (41)

where *φ*_c1_, *φ*_c2_ are the phases at which the transition occurs, and are given by eq (14). Consequently, the phase of the input profile is *ψ* = (*φ*_c1_, *φ*_c2_)/2, and the self-consistent: eq (40) is reduced to a self-consistent condition on the phases

$$0=\frac{\varphi_{c1}+\varphi_{c2}}{2}+\nu d,$$ (42)

where we have taken, without loss of generality, the phase of the post-synaptic neuron to be zero. Thus, for example, the temporally anti-symmetric case (exponential kernel of eq (4) with *τ*_+_ = *τ*_−_), with *α* = 1 yields *φ*_c1_ = −*π* and *φ*_c2_ = 0, resulting in the condition *ν*d = *π*/2 for zero drift. Fig 5A shows the drift velocity as a function of the angular frequency of the oscillations *ν*/(2*π*) for a conductance based integrate and fire post-synaptic neuron. As can be seen from the figure, the drift velocity vanishes at a frequency *f* ≈ 29*Hz*, which is consistent with a delay *d* ≈ 8.6*ms* that can be measured numerically from the phase difference between the post-synaptic neuron and its input.

**Fig 6:**
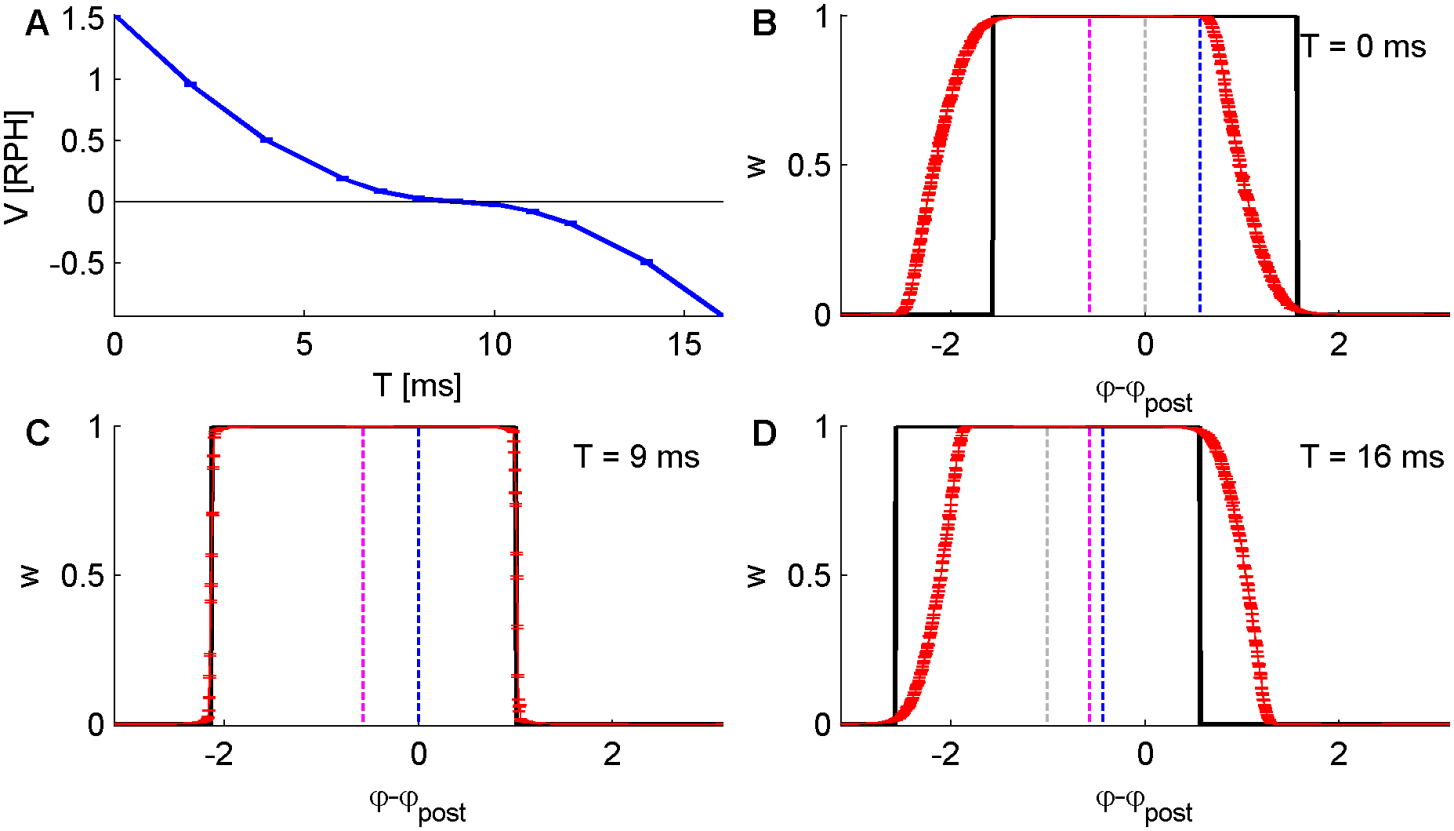
Numerical results of simulations as in Fig 5, with pre-synaptic activity following eq (17), with *N* = 1200, *D* = 10*Sp*/sec, *A* = 10*Sp*/sec, *ν* = 2*π*·10*Hz*. The simulated STDP rule was the temporally shifted “Mexican hat” kernel (as in Fig 1D) with varying *T*_±_ = *T*, and with *α* = 1, *μ* = 0. (A) The angular velocity (*V* of eq (34)–in Revolutions per Hour—RPH) as a function of the time shift *T*. (B, C and D) show the same plots as in Fig 5 for 3 of the simulations with *T* = 0,9,16*ms*, respectively. Here *λ* = 5·10^−3^ was used.

In the example of a temporally shifted Mexican hat learning rule (difference of Gaussians kernel with *T*_+_ = *T*_−_ ≡ *T*,) *φ*_c1,2_ = −*νT* ± *β*; hence, *Ψ* = −*νT*. Thus, the condition for zero drift velocity is *T* = *d*. Fig 6A shows the drift velocity as a function of *T* under this learning rule, for a conductance-based integrate and fire post-synaptic neuron. As seen from the figure, the drift velocity vanishes at a delay d ≈ 9*ms*.

Note that due to the non-linearity of the post-synaptic neuron and the conductance response of the synapses (see Methods), the evaluated delay between pre- to post- synaptic activities is not exactly the same in the two examples, which mainly differ by the oscillation frequency. Nevertheless, under these zero drift conditions the steady state of the linear solution given by eq (41) provides a good approximation of the measured profile, see e.g., Figs 5C and 6C. Part of the reason for such minor influences of this non-linearity is that higher order Fourier modes of the post-synaptic activity are orthogonal to the pre-synaptic activity.

How can one understand intuitively the source of this drifting behavior? Consider the case of a temporally symmetric Mexican hat STDP rule (eq (5) with *T*_+_ = T_−_: = *T* = 0, red line in Fig 1D). This rule potentiates synapses with a similar phase preference as the post-synaptic neuron see e.g. Fig 2A2. Let us assume that by some mechanism of spontaneous symmetry breaking the synaptic weight profile develops a preferred phase, and without loss of generality assume *ψ* = 0, the vertical gray dashed line in Fig 6B. Consequently, the post-synaptic neuron will oscillate with a phase *φ*_*post*_ = *ψ* + *dν*, the vertical blue dashed line in Fig 6B. This in turn will cause the synaptic dynamics to generate a ‘force’ that will pull the peak of the weight profile, *ψ*, towards *φ*_*post*_ = *ψ* + *dν* and hence, will induce a positive drift velocity.

## Discussion

STDP is known to be able to act as an unsupervised learning algorithm that learns prominent features of the input layer (pre-synaptic population) statistic; namely, the spatial structure of the correlations. Via a local synaptic STDP learning rule, spatial (or stimulus) selectivity can emerge [14, 15, 17, 20, 47–49]. Here, we focused on temporal aspects and showed how temporal selectivity may emerge. Specifically, we found that although the net activity of the input layer (pre-synaptic population) was constant in time, the homogeneous state was not always stable and the post-synaptic neuron developed temporal phase preferences via a mechanism of spontaneous symmetry breaking. This instability depends on the sign of the real part of the Fourier transform of the STDP rule (potentiation minus depression) which is time shifted by the post-synaptic delay *d* at angular frequency *ν* (see eq (33)). However, in contrast to previous studies, we found that in many cases this selectivity is not static, but rather it drifts in time.

One can view Fig 6B as a graphic illustration of a search for a self-consistent solution with zero drift. Assume that the post-synaptic neuron oscillates at some constant phase, without loss of generality *φ*_*post*_ = 0. The STDP will shape the synaptic weight profile symmetrically around *φ*_*post*_ according to eq (40), the black line in Fig 6B. Accordingly, the synaptic weight profile and the *input* to the post synaptic neuron will have a phase of *ψ* = *φ*_*post*_ = 0, the vertical dashed gray line in Fig 6B. However, the response of the post neuron to its inputs dictates *φ*_*post*_ = *ψ* + *dν* (shown by the vertical blue line), which is inconsistent with our initial assumption *φ*_*post*_ = 0. In order to satisfy the self-consistent condition we need to introduce a temporal shift to the STDP rule (eq (5) with *T*_+_ = *T*_−_ ≡*T ≠* 0) that will align the initial assumption of the post phase at *φ*_*post*_ = 0 with *ψ* + *dν*, Fig 6C. Further increases in the temporal shift *T* of the STDP rule will generate a ‘force’ that will pull the weight profile to the left and will result in a negative drift velocity, see Fig 6D.

A quasi-periodic behavior of synaptic weights has been observed in the past. Gilson and colleagues [48], studied the effect of general correlation structure on STDP-driven synaptic dynamics. Analyzing the homogeneous fixed point they assumed the synaptic dynamics will flow in the direction of the strongest spectral component (of the input layer correlations), and consequently will converge to a stable fixed point that will reflect the structure of the spectral component. In the additive learning rule (i.e., *μ* = 0) or in the case where the neuronal response covariance is negative the existence of a stable fixed point is not guaranteed. Thus, it was shown that for the special case of additive STDP synaptic dynamics may be dominated by eigenvalues with large imaginary part that will result in quasi periodic behavior. This pathology disappears for any positive *μ*.

Here, the limit cycle solution is not a pathology but a robust feature of the synaptic dynamics (see for example limit cycle solution with *μ* > 0 in Fig 4). The main difference from the work of Gilson and colleagues is that, typically, here there is no stable fixed point solution (note that response covariance of input layer neurons can be negative). Even in the special case where a non-homogeneous fixed point exists it is: One, Not generic but requires a set of parameters that will solve *V* = 0. Two, due to the inherent *U*(1) symmetry of the problem this solution will be only marginally stable.

Theoretical investigations of STDP typically derive non-linear equations for the dynamics of the synaptic weights. In general, non-linear dynamics is known to give rise to a wide range of behaviors, including convergence to a fixed point, line attractors, oscillatory activity, and chaos. However, to the best of our knowledge, the existing theoretical research describes STDP as a process that relaxes to a steady state fixed point (except for pathological cases). On the other hand, empirical findings show synaptic dynamics as highly volatile and even chaotic [50–58]. This raises the question of how can the central nervous system retain its functionality in the face of constant remodeling of synaptic weights? Here we have shown an example in which synaptic weights did not converge to a stable fixed point, but rather remained dynamic. Yet, functionality, in this case oscillating activity of the post-synaptic neuron, was maintained as an emergent property of global order parameters $\bar{w}$ and $\left|\tilde{w}\right|$ that converge to a stable fixed point.

## Methods

### Details of the numerical simulations

#### Online supporting information

This manuscript is accompanied by a complete software package that was used throughout the study. This package is a Matlab set of scripts and utilities that includes all the source codes for numerical simulations that were used to produce the figures in this manuscript. It also contains all the scripts that generated the figures.

#### The conductance based integrate-and-fire model

The learning dynamics, eq (1), were simulated using a conductance based integrate-and-fire post-synaptic cell. Details of the simulations are as in previous studies [25, 26]. Briefly, the membrane potential of the post-synaptic cell *V*(*t*) obeys

$$C_{m}\frac{dV}{dt}=\frac{\left(V_{rest}-V\right)}{R_{m}}+g_{E}\left(E_{E}-V\right)+g_{I}\left(E_{I}-V\right),$$ (43)

where *C*_*m*_ = 200pF is the membrane capacitance, *R*_*m*_ = 100MΩ is the membrane resistance, the resting potential is *V*_*rest*_ = −70mV, and the reversal potentials are *E*_*E*_ = 0mV and *E*_*I*_ = −70mV. An action potential is generated once the membrane potential crosses the firing threshold *V*_*th*_ = −54*mV*, after which the membrane potential is reset to the resting potential without a refractory period.

The synaptic conductances, *g*_*E*_ and *g*_*I*_, are given by:

$$g_{X}\left(t\right)=g_{X}^{0}\overset{N_{X}}{\underset{i=1}{\sum }}\left(w_{i}^{X}\left(t\right)\underset{j}{\sum }{\left[t-t_{j}^{i}\right]}_{+}e^{-\left(t-t_{j}^{i}\right)/\tau_{X}}\right),$$ (44)

where *X = E*, *I* denotes excitatory or inhibitory nature, *N*_*X*_ is the number of synapses, [*t*]_+_≡*max*(t,0) and is measured in seconds, and ${\left\{t_{j}^{i}\right\}}_{j}$ are the spike times at synapse *i*. For the temporal characteristic of the α-shape response we chose to use *τ*_*E*_ = *τ*_*I*_ = 5*ms*. For the conductance coefficient $g_{X}^{0}$ we used the normalization factor of 1/N in eq (19). We used $g_{X}^{0}=g_{X}^{R}S_{X}$ with $g_{E}^{R}=30nS$, *S*_*E*_ = 1000/*N*_*E*_, $g_{I}^{R}=50nS$ and *S*_*I*_ = 400/*N*_*I*_, where *N*_*E*_, *N*_*I*_ denote the number of excitatory and inhibitory pre-synaptic inputs, respectively.

In order to estimate the post-synaptic membrane potential in eq (43), the software performs the integration of the synaptic and leak currents using the Euler method with a Δ*t* = 1*ms* step size. The rationale for using such a low resolution step size is justified in our previous work [26].

#### Modeling pre-synaptic activity

Throughout the simulations in this work, pre-synaptic activities were modeled by independent inhomogeneous Poisson processes, with a modulated mean firing rate given by eq (17). To this end, each of the inputs was approximated by a Bernoulli process generating binary vectors defined over discrete time bins of Δ*t* = 1*ms*. These vectors were then filtered using a discrete convolution α-shaped kernel (as defined by eq (44)) with a limited length of 10*τ*_*X*_ (after which this kernel function is zero for all practical purposes). In all simulations we used *N*_*I*_ = 40, and selectively used *N*_*E*_ ∈ {120, 1200} as expressed in the different figures. Note that the synaptic conductances are scaled inversely with the number of synapses.

## Supporting Information

S1 Software A Matlab set of scripts and utilities that includes all the source codes for numerical simulations that were used to produce the figures in this manuscript. It also contains all the scripts that generated the figures.
(ZIP)
